# Vitamin D Reverses Disruption of Gut Epithelial Barrier Function Caused by *Campylobacter jejuni*

**DOI:** 10.3390/ijms22168872

**Published:** 2021-08-18

**Authors:** Fábia D. Lobo de Sá, Steffen Backert, Praveen K. Nattramilarasu, Soraya Mousavi, Geoffrey I. Sandle, Stefan Bereswill, Markus M. Heimesaat, Jörg-Dieter Schulzke, Roland Bücker

**Affiliations:** 1 Nutritional Medicine/Clinical Physiology, Medical Department, Division of Gastroenterology, Infectious Diseases, Rheumatology, Charité—Universitätsmedizin Berlin, 12203 Berlin, Germany; fabia.lobo-da-fonseca@charite.de (F.D.L.d.S.); praveen-kumar.nattramilarasu@charite.de (P.K.N.); joerg.schulzke@charite.de (J.-D.S.); 2 Division of Microbiology, Department of Biology, University of Erlangen-Nürnberg, 91058 Erlangen, Germany; Steffen.Backert@fau.de; 3 Institute of Microbiology, Infectious Diseases and Immunology, Charité—Universitätsmedizin Berlin, 12203 Berlin, Germany; soraya.mousavi@charite.de (S.M.); stefan.bereswill@charite.de (S.B.); markus.heimesaat@charite.de (M.M.H.); 4 Institute of Medical Research at St James’s, St James’s University Hospital, Leeds LS9 7TF, UK; G.I.Sandle@leeds.ac.uk

**Keywords:** tight junction, campylobacteriosis, apoptosis, translocation, leaky gut, vitamin D receptor signaling pathway, RNA Sequencing, mouse model, HT-29/B6 cell, intestinal epithelial barrier function

## Abstract

Infections by the zoonotic foodborne bacterium *Campylobacter jejuni* (*C. jejuni*) are among the most frequent causes of bacterial gastroenteritis worldwide. The aim was to evaluate the relationship between epithelial barrier disruption, mucosal immune activation, and vitamin D (VD) treatment during *C. jejuni* infection, using intestinal epithelial cells and mouse models focused on the interaction of *C. jejuni* with the VD signaling pathway and VD treatment to improve *C. jejuni*-induced barrier dysfunction. Our RNA-Seq data from campylobacteriosis patients demonstrate inhibition of VD receptor (VDR) downstream targets, consistent with suppression of immune function. Barrier-preserving effects of VD addition were identified in *C. jejuni*-infected epithelial cells and IL-10^−/−^ mice. Furthermore, interference of *C. jejuni* with the VDR pathway was shown via VDR/retinoid X receptor (RXR) interaction. Paracellular leakiness of infected epithelia correlated with tight junction (TJ) protein redistribution off the TJ domain and apoptosis induction. Supplementation with VD reversed barrier impairment and prevented inhibition of the VDR pathway, as shown by restoration of transepithelial electrical resistance and fluorescein (332 Da) permeability. We conclude that VD treatment restores gut epithelial barrier functionality and decreases bacterial transmigration and might, therefore, be a promising compound for *C. jejuni* treatment in humans and animals.

## 1. Introduction

The most common cause of bacterial gastroenteritis worldwide is the foodborne zoonotic bacterium *Campylobacter jejuni* (*C. jejuni*). *C. jejuni* is an intestinal commensal in a variety of animals (particularly in poultry), but after fecal–oral transmission is pathogenic in the human intestine, causing an acute infective colitis. Campylobacteriosis is characterized by watery, bloody diarrhea, abdominal cramps, fever, and intestinal inflammation [1]. The infection is usually self-limiting and only symptomatic treatment is required, but chronic progression or infections in immunocompromised patients require antibiotic treatment. A proportion of campylobacteriosis patients develop sequelae such as Guillain–Barré syndrome, reactive arthritis, or irritable bowel syndrome (IBS). Studies in *C. jejuni*-infected volunteers indicated that an acute inflammatory response is a hallmark of the infection [1]. Experimental studies revealed a strong interrelation between the mucosal immune response and epithelial barrier dysfunction in *C. jejuni* infection: the ‘leaky gut’ phenomenon [2,3,4].

The pre-hormone vitamin D (VD) regulates innate and adaptive immune responses. Its precursor cholecalciferol (calciol; (3β,5Z,7E)-9,10-secocholesta-5,7,10(19)-trien-3-ol) is metabolized to the intermediate calcidiol (25-hydroxycholecalciferol) and then to biologically active vitamin D3 (calcitriol; 1,25-dihydroxycholecalciferol). Low serum VD levels increase susceptibility to acute and chronic illnesses such as infectious diseases, IBS, inflammatory bowel disease (IBD), and colorectal cancer [5,6,7,8]. VD is well known for its role in bone metabolism, but is also involved in regulating the absorptive and immune functions of the gastrointestinal tract [9]. In the intestine, vitamin D is required for cell differentiation, proliferation, and for the maintenance of epithelial barrier function, in addition to its effects on calcium and phosphate transport [10]. The biological active form of VD is hydroxylated to calcitriol by 1-α-hydroxylase (also known as cytochrome P450 enzyme CYP27B1) and regulates further target genes in a complex with the VD receptor (VDR) and the retinoid X receptor (RXR). Evidence for the barrier-strengthening potential of VD has come from studies in IBD [11]. VD also exhibited barrier-protective and anti-inflammatory properties in in vitro and in vivo studies, but without a clear explanation for its molecular mechanism of action [12,13,14,15,16,17]. Nevertheless, clinical studies have shown a beneficial effect of VD treatment on diarrhea in IBD patients [18], which raises the possibility that it might also improve bacteria-induced diarrhea.

In this regard, VD supplementation increased the viability of intestinal epithelial cells, and partially restored the reduced transepithelial electrical resistance (TER) that occurred during *C. jejuni* infection [2]. Furthermore, in a preclinical intervention mouse study, VD decreased the inflammatory response that occurred during campylobacteriosis [14]. In the present study, we built on our previous work using bioinformatics to identify the relevance of the VD pathway in campylobacteriosis [2] by investigating (i) whether *C. jejuni*-induced barrier dysfunction can be treated or prevented by VD supplementation, and (ii) how *C. jejuni* interferes with the VD pathway.

## 2. Results

### 2.1. Upstream Regulator Analysis of C. jejuni—Infected Human Colonic Mucosa and VDR Downstream Targets

**Figure 1:**
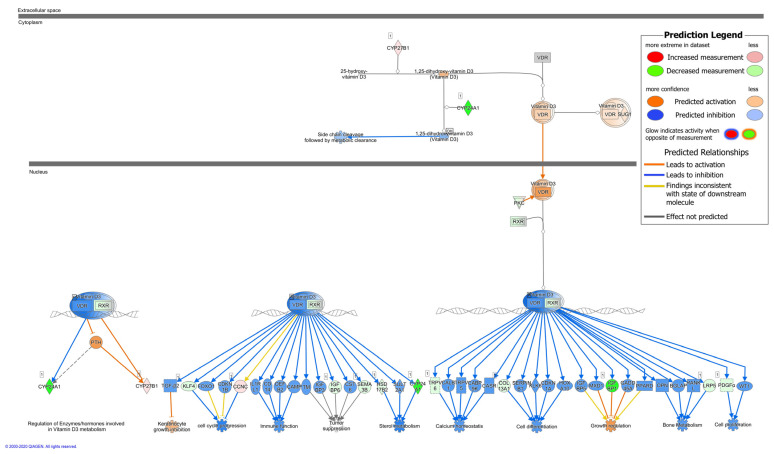
Ingenuity Pathway Analysis (IPA) of RNA-Seq data showing VDR/RXR activation in colonic mucosae from *C. jejuni*-infected patients. CYP27B1 activates 25-hydroxy vitamin D3 (calcidiol) to biologically active 1,25-dihydroxy-vitamin D3 (calcitriol). CYP24A1 inactivates vitamin D and therefore opposes the action of CYP27B1. Active vitamin D binds to the VD receptor (VDR), enters the nucleus, forms a complex with the retinoid X receptor (RXR), and influences the expression of different target genes. Blue arrows indicate predicted inhibition and orange arrows predict activation of the network components. A colored legend is shown in the upper right corner of the figure.

Our RNA-Seq analysis of colonic mucosa from *C. jejuni*-infected patients predicted inhibition of the ability of vitamin D (VD) to act as an upstream regulator [2]. Examination of this analysis in detail revealed that active VD (calcitriol) had a negative activation Z-score of −6.3 with an overlap *p*-value of 8.97 × 10^−5^. This highly significant negative Z-score with inhibited VDR pathways suggested that administration of VD might counter-regulate inhibited VD downstream targets and protect the host intestine from *C. jejuni* infection. Based on the RNA-Seq data from colonic mucosa of *C. jejuni*-infected patients, activity patterns of vitamin D receptor (VDR)/retinoid X receptor (RXR) pathways were analyzed using Ingenuity Pathway Analysis (IPA) software, which demonstrated that downstream targets related to immune regulation, cell proliferation, and cell differentiation in the patients’ colon mucosae were largely inhibited during campylobacteriosis (Figure 1).

For example, in the case of downstream VDR/RXR pathways regulating immune function, target genes for beta-defensin 2 (*DEFB2*, *DEFB4*), cathelicidin (*CAMP*), CD14 (*CD14*) and IL-1 receptor (*IL1RL1*, activated by IL-1β), were inhibited during *C. jejuni* infection as indicated (Figure 1, blue arrows). During infection, most downstream targets should show activation of their respective pathways (Figure 1, orange arrows). This has led to the hypothesis that *C. jejuni* directly interferes with the VDR pathway, which is targeted for activation in other infections [19].

### 2.2. Epithelial Barrier Dysfunction in C. jejuni Infection Is Reversed by Vitamin D

**Figure 2:**
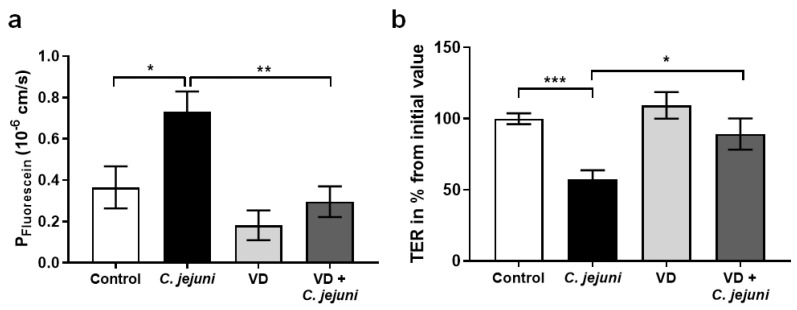
Vitamin D inhibits *C. jejuni*-induced barrier dysfunction in vitro. HT-29/B6-GR/MR cells grown on filter supports were pre-treated with or without 200 nM vitamin D (VD) from the apical and basolateral sides and infected with *C. jejuni* from the apical side with a multiplicity of infection (MOI) of 100. After 48 h microaerobic incubation (**a**) 332 Da fluorescein permeability between the apical and basolateral compartments was determined (* *p* < 0.05, ** *p* < 0.01, one-way ANOVA with Bonferroni correction, *n* = 6–7) and (**b**) transepithelial electrical resistance (TER) was measured using chopstick electrodes (* *p* < 0.05, *** *p* < 0.001, one-way ANOVA with Bonferroni correction, *n* = 13–14).

Based on RNA-Seq data from campylobacteriosis patients, the predicted potential effectiveness of active VD against the pathophysiological changes induced by *C. jejuni* was investigated in more detail using different in vitro and in vivo approaches. In in vitro studies, *C. jejuni* increased the paracellular permeability of HT-29/B6-GR/MR cell monolayers to fluorescein (332 Da) (Figure 2a). In the presence of VD, permeability to fluorescein was even lower than in controls. Moreover, *C. jejuni* decreased transepithelial electrical resistance (TER), reflecting an increase in ionic permeability (Figure 2b). These changes point to disruption of barrier function in the intestinal epithelial cell monolayers. However, the presence of VD in *C. jejuni*-infected monolayers prevented the barrier-disrupting effect of the bacterium, and TER and epithelial permeability were restored to control levels (Figure 2).

**Figure 3:**
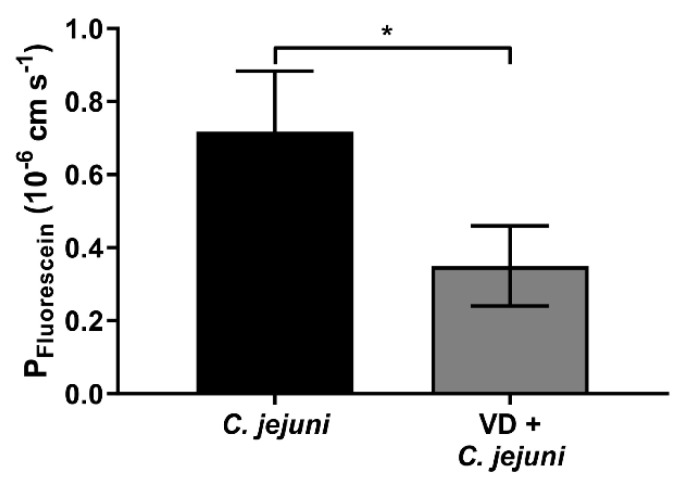
Vitamin D treatment prevents *C. jejuni*-induced barrier dysfunction in vivo. Secondary abiotic IL-10^−/−^ mice were orally infected with 10^8^ colony forming units (CFU) *C. jejuni.* Cholecalciferol was administered via the drinking water *ad libitum* four days prior the infection with *C. jejuni*. Six days after infection with *C. jejuni*, mice were scarified, colonic biopsies were mounted immediately in miniaturized Ussing chambers, and fluorescein (332 Da) permeability was measured from the apical to basolateral compartment (* *p* < 0.05, Mann–Whitney U-test, *n* = 7–14).

In order to obtain confirmatory evidence in an in vivo ‘leaky gut’ mouse model, pre-conditioned IL-10^−/−^ mice were infected with *C. jejuni* and treated with cholecalciferol added to their drinking water. Animals receiving cholecalciferol showed a lower permeability to 332 Da fluorescein compared to placebo-treated *C. jejuni*-infected mice (Figure 3). In accordance with 3R principles guidelines, the number of animals studied was minimized by using animals from a collaborative study [14].

### 2.3. Influence of C. jejuni and Vitamin D on the Protein Expression and Distribution of Tight Junction Proteins

Intestinal epithelial barrier dysfunction correlates with changes in the expression or subcellular distribution of tight junction (TJ) proteins. Protein expression of different barrier-relevant TJ proteins in epithelial monolayers was quantified by densitometric analysis of Western blots, and revealed that *C. jejuni* infection caused no decreases in the expression of the sealing TJ proteins claudin-4, -5, -8, and the TJ proteins claudin-7, or occludin (Table 1).

**Figure 4:**
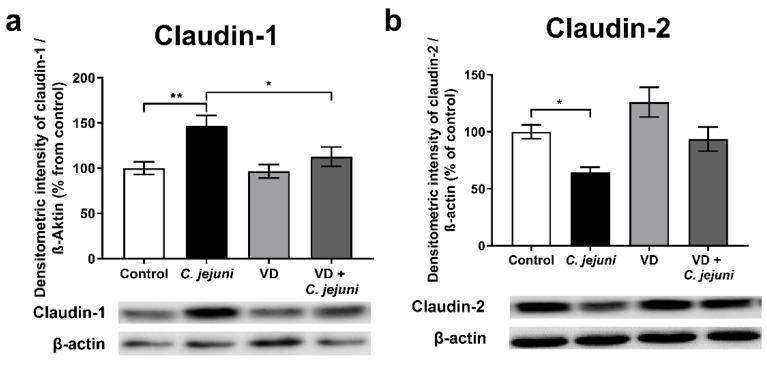
Tight junction protein expression changes in vitamin D-treated *C. jejuni*-infected epithelial cells. *C. jejuni*-infected HT-29/B6-GR/MR cells were treated with 200 nM vitamin D. Cells were lysed 48 h after infection and changes in (**a**) claudin-1 and (**b**) claudin-2 determined by Western blot analysis and normalized to β-actin (* *p* < 0.05, ** *p* < 0.01, one-way ANOVA with Bonferroni correction, *n* = 9–10).

*C. jejuni* infection also increased the expression of the barrier-forming TJ protein claudin-1 (Figure 4a) and decreased that of channel-forming claudin-2 (Figure 4b) but neither of these changes could account for *C. jejuni*-induced barrier impairment. VD inhibited the *C. jejuni*-induced changes in claudin-1 expression, which also does not explain the changes in the barrier function induced by infection.

**Figure 5:**
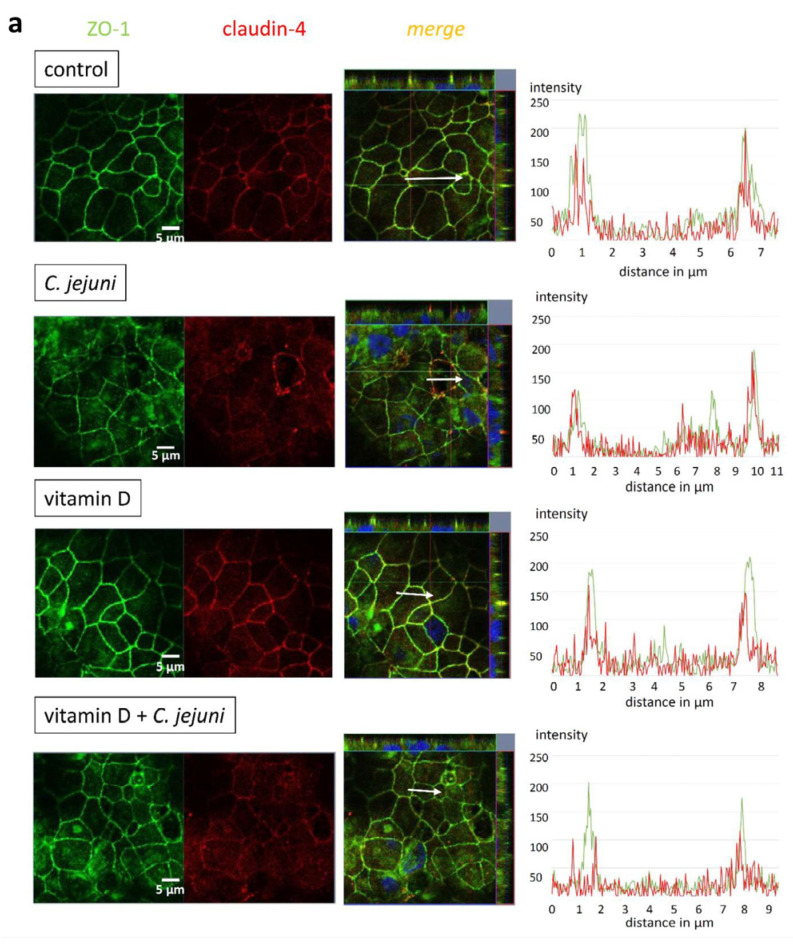

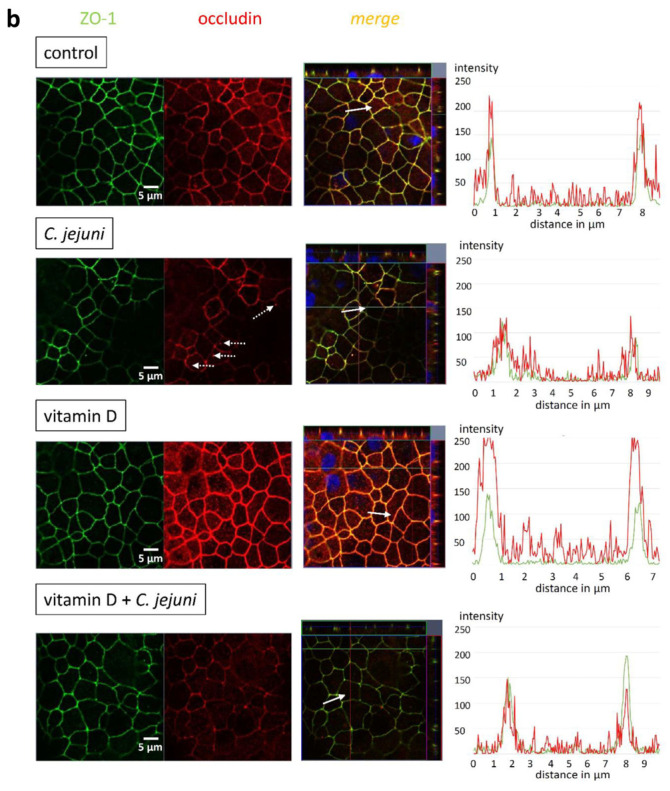
*C. jejuni*-induced redistribution of tight junction proteins is prevented by vitamin D treatment in vitro. *C. jejuni*-infected HT-29/B6-GR/MR cells were treated with 200 nM vitamin D. 48 h after infection with *C. jejuni*, cells were stained with antibodies to ZO-1 (green) and (**a**) claudin-4 (red) or (**b**) occludin (red), and visualized by confocal laser-scanning microscopy. Nuclei were stained blue with 4′,6-diamidino-2-phenylindole (DAPI) (bar 5 µm). Co-localization of the TJ proteins is depicted by a yellow merge signal. White arrows in the z-stacks indicate the analyzed area of the displayed intensity distance plot. Intensity distance plots indicate changes in the signal intensity on the abscissa, and redistribution to intracellular regions on the ordinate.

*C. jejuni*-infected epithelial monolayers, either treated with VD or not, were immunostained for different barrier-relevant TJ proteins and visualized with confocal laser-scanning microscopy (CLSM). Microscopic signals from TJ proteins claudin-4 (Figure 5a) and occludin (Figure 5b) showed that the TJ domain was redistributed into intracellular compartments after *C. jejuni* infection, and both were less co-localized with zonula occludens protein-1 (ZO-1), changes consistent with impaired epithelial barrier function. This *C. jejuni* infection-induced redistribution of TJ proteins was inhibited by VD treatment (Figure 5) in keeping with the ability of VD to restore barrier function normal levels.

### 2.4. Vitamin D Prevents C. jejuni—Induced Epithelial Apoptosis

**Figure 6:**
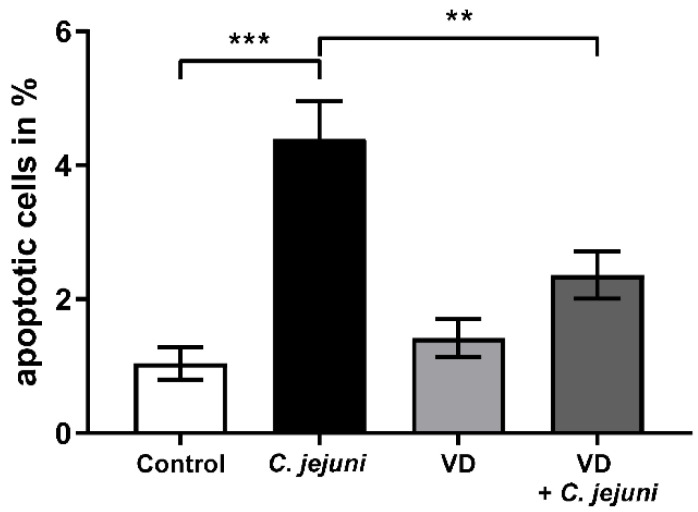
Vitamin D prevents epithelial cells from *C. jejuni*-induced apoptosis. *C. jejuni*-infected HT-29/B6-GR/MR cells were treated with 200 nM vitamin D (VD). After 48 h of infection, cells were stained for apoptotic cells with TUNEL reagent, and visualized by confocal laser-scanning microscopy. Nuclei were stained blue with 4´,6-diamidino-2-phenylindole (DAPI) and counted in relation to TUNEL-positive signals. The number of apoptotic cells was estimated in six low-power fields per sample (** *p* < 0.01, *** *p* < 0.001, one-way ANOVA with Bonferroni correction, *n* = 4).

As the observed changes in TJ protein expression and distribution may not have been the only cause of barrier dysfunction in the epithelial cell monolayers, the role of apoptosis was analyzed by fluorescence microscopy and TdT-mediated dUTP-biotin nick end labeling (TUNEL) assay. *C. jejuni* infection increased the apoptosis ratio, which was prevented by VD treatment (Figure 6).

### 2.5. Vitamin D Supplementation Reduces the Number of Transmigrated C. jejuni

**Figure 7:**
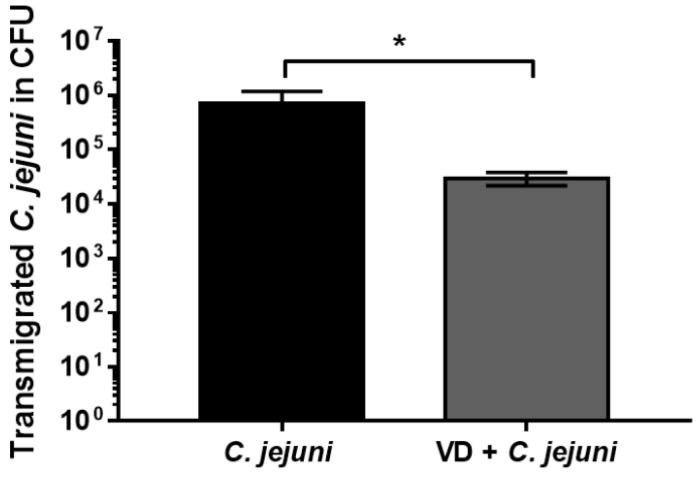
Transmigration of *C. jejuni* across intestinal epithelial cell monolayers. HT-29/B6-GR/MR cells were treated with 200 nM vitamin D and infected with *C. jejuni* from the apical side. Eight hours after infection, bacteria that had transmigrated into the basolateral compartment were collected from the bathing medium and quantified by counting CFU on blood agar plates (* *p* < 0.05, Mann–Whitney U-test, *n* = 11–16).

*C. jejuni* invades the intestinal epithelium, transmigrating from the lumen to the underlying immune cells, resulting in activation of the innate immune response. In our HT-29/B6 cell culture system, VD inhibited bacterial transmigration 8 h after infection with *C. jejuni* (Figure 7).

In addition, the presence of 200 nM VD inhibited bacterial transmigration between MKN-28 cells after infection from 3.4 ± 0.5 × 10^7^ CFU to 1 ± 0.8 × 10^5^ CFU (*n* = 3, *p*-value = 0.000645). These observations indicate that VD inhibited the development of barrier dysfunction, thereby maintaining barrier functionality, and thus minimizing bacterial translocation. 

### 2.6. Influence of C. jejuni on the Vitamin D Pathway and Its Functional Importance

**Figure 8:**
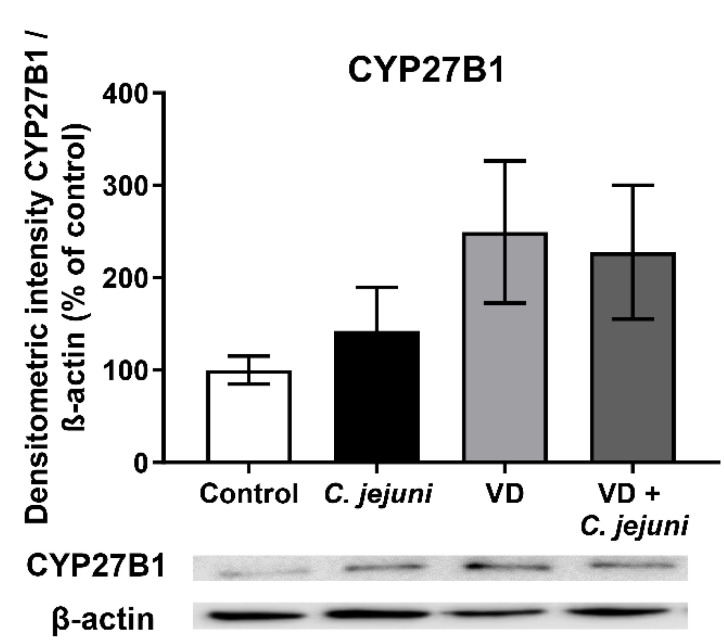
*C. jejuni* influences CYP27B1 protein expression. HT-29/B6-GR/MR cells were infected with *C. jejuni* from the apical side with an MOI 100 and treated with active vitamin D (VD). Cells were lysed 48 h after infection with *C. jejuni*, and changes in CYP27B1 protein expression analyzed by Western blot and normalized to β-actin. There were no significant differences between the groups (one-way ANOVA with Bonferroni correction, *n* = 9–10).

Using IPA, upstream regulator analysis of colon mucosae from *C. jejuni*-infected patients revealed inhibited activation states of the VDR (negative Z-score −3.2, *p* = 1 × 10^−4^) and CYP27B1 (negative Z-score −2.4, *p* = 5.5 × 10^−3^). However, RNA-Seq data indicated that *VDR* gene expression was unchanged, while that of *CYP27B1* was increased (Figure 1). Similar results for CYP27B1 were obtained in Western blot analyses of *C. jejuni*-infected human HT-29/B6-GR/MR intestinal epithelial cells (Figure 8). CYP27B1 protein expression was not significantly different between *C. jejuni*-infected epithelial cells and controls, although it tended to increase during infection, particularly in the presence of VD.

**Figure 9:**
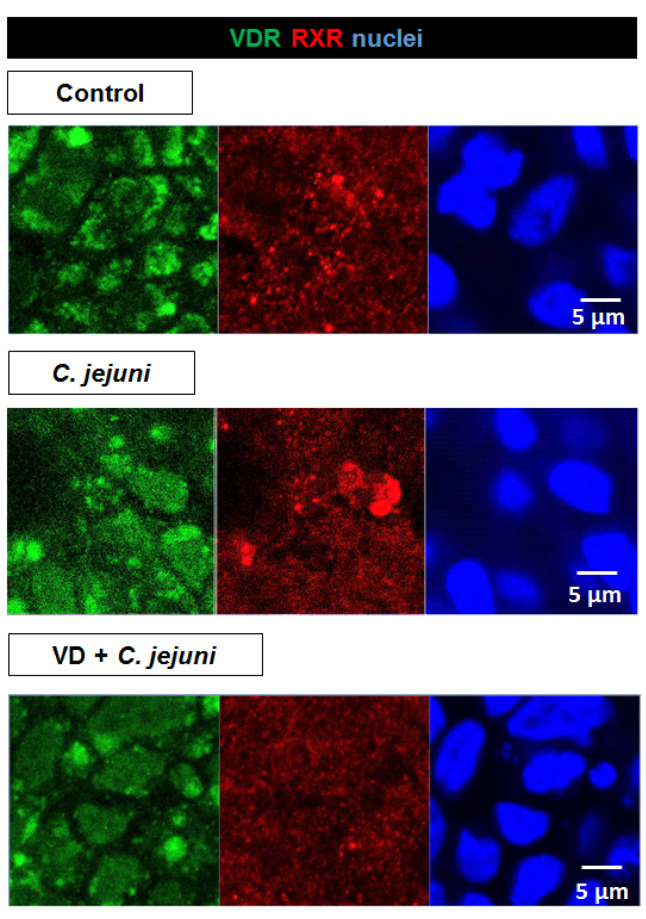
Changes in the localization of the VDR/RXR complex in epithelial cells after *C. jejuni* infection and vitamin D (VD) treatment. After 48 h of infection with *C. jejuni* and treatment with VD, HT-29/B6-GR/MR cells were immunostained with antibodies to the vitamin D receptor (VDR; green), and the retinoid-X receptor (RXR; red), and nuclei were stained blue with 4′,6-diamidino-2-phenylindole (DAPI). Changes in localization and intensity of VDR-RXR staining were determined by confocal laser-scanning microscopy (bar 5 µm).

VDRs are normally located within the nuclei. However, in *C. jejuni*-infected cells, VDR signals moved from the nucleoplasm to cytosolic compartments and were re-localized to the nuclei after VD treatment (Figure 9).

In addition, we used CLSM to analyze the VDR/retinoid X receptor (RXR) complex, VDR/RXR heterodimers being generally present in both cytoplasm and nucleus, with a greater amount in the nucleus [20]. Interestingly, fluorescence signals from RXR were more condensed (i.e., accumulated) in the *C. jejuni*-infected group than in the controls (Figure 9). VD treatment of *C. jejuni*-infected cells inhibited this condensation of the VDR/RXR complexes, so that the distribution of VDR/RXR complexes was similar to that seen in uninfected untreated controls (Figure 9). Condensation of VDR/RXR in the *C. jejuni*-infected group suggests strongly that *C. jejuni* infection interferes with downstream VDR signaling, leading to inhibition of downstream target genes and functional inhibition of their DNA transcripts, as predicted by IPA in the RNA-Seq data from the colon mucosae of *C. jejuni*-infected patients (Figure 1).

## 3. Discussion

Vitamin D status has an impact on the pathogenesis of IBD, colorectal cancer [5,6,7], and acute enteric infections such as that produced by *Escherichia coli* [8,21,22]. This suggests that a VD therapy in the form of cholecalciferol or calcitriol may have a place in the management of some common intestinal infections, and patients have been treated previously with doses ranging from 1800 to 10,000 international units (IU) per day [18,23]. VD status has also been shown to influence the disease outcome of corona virus (COVID-19) infection in risk groups [24]. As *C. jejuni* infections are a major clinical problem, we questioned whether VD might also influence the pathogenesis of *C. jejuni* enteritis and, if so, whether VD might have a positive impact on the prevention and/or treatment of this type of intestinal infection. To date, our previous studies have established that VD has a role in *C. jejuni*-induced barrier dysfunction [2,14] without identifying the mechanisms underlying its protective properties. 

### 3.1. Effect of C. jejuni and Vitamin D on Tight Junction Protein Expression, Localization, and Apoptosis Induction

Previous studies have demonstrated epithelial barrier disturbance, with decreased TER and increased permeabilities for high molecular weight paracellular markers in *C. jejuni*-infected in vitro cell lines, in vivo mouse models, and colonic mucosae from *C. jejuni*-infected patients. In all of these models, barrier dysfunction reflected a combination of changes in TJ protein expression and localization and the induction of apoptosis [2,3,4,14]. Similarly, in the present study in *C. jejuni*-infected HT-29/B6 cells, we observed a decrease in TER and an increased permeability to fluorescein (332 Da), and both changes were reversed by VD. VD has also been shown to restore impaired barrier function induced by ethanol [12], TNF-α [13,25], and dextran sulfate sodium (DSS) [26,27] in other in vitro models. 

Using *C. jejuni*-infected IL-10^−/−^ mice as an in vivo ‘leaky gut’ model, high-dosage cholecalciferol added to their drinking water reversed the infection-induced increase in colonic permeability to fluorescein, confirming the results of our in vitro experiments. The restorative effects of cholecalciferol on the permeability of larger molecules such as FITC-dextran (4 kDa) have also been reported in DSS and 2,4,6-trinitrobenzenesulfonic acid (TNBS)-induced mouse model of colitis [25,27]. In colon mucosae of *C. jejuni*-infected IL-10^−/−^ mice treated with cholecalciferol, TER was similar to that in control mice 6 days post-infection, and this effect of cholecalciferol was associated with reduced intestinal and extra-intestinal inflammatory responses, and a decrease in innate immune cells as well as diminished diarrheal frequency during the development of campylobacteriosis [14]. We speculate that calcitriol administration may have an even greater beneficial effect.

Disruption of the epithelial barrier function reflects changes in the expression and/or localization of TJ proteins. As in other models, expression of the sealing TJ protein claudin-1 increased during *C. jejuni* infection, despite the decrease in TER. This is called the ‘claudin-1 paradox’, and is due to the disassembly of claudin-1 away from the TJ domain [2]. Further mechanisms involve trafficking of TJ components or disruption of the perijunctional actomyosin cytoskeleton such as F-actin reorganization as shown, e.g., for *Aeromonas hydrophila* toxin, where TJ proteins were retraced from the TJ domain by actomyosin constriction or caveolin-dependent endocytosis of intrajunctional occludin [28,29,30]. In addition, *C. jejuni* downregulates the water- and cation-channel TJ protein claudin-2, which shows some increase after VD treatment and, in our model, may represent counter-regulation. Claudin-2 gene expression is a direct target of the VDR and is upregulated via the VDR promotor binding site VDRE [15]. Interestingly, recent studies demonstrated a link between downregulation of VDR and overexpression of *CLDN2* in *Salmonella* infection [16,31]. Redistribution of occludin and of the sealing TJ protein claudin-4 induced by *C. jejuni* contributes to barrier dysfunction by opening the paracellular leak pathway to ions, water, and macromolecules, so that antigen influx activates immune cells and triggers cytokine release, which further compromises the epithelial barrier and results in a ‘leaky gut’. In our in vitro model, VD restored, at least in part, the expression and normal distribution of the TJ proteins, as reported in TNF-α-stimulated Caco-2 cells [25] and in T84 cells or in colonic biopsies from patients with ulcerative colitis [32].

Apoptosis also contributes to epithelial leakiness and the ‘leaky gut’. The *C. jejuni*-induced apoptosis contributes to epithelial barrier dysfunction as shown in a previous study, with the pan-caspase inhibitor Q-VD-OPh, which restores the decreased TER and the increased permeability to fluorescein (332 Da) and FITC-dextran (4 kDa) [4]. Due to its anti-tumorigenic and either pro- or anti-apoptotic effects in different cell types, VD is commonly used as a supplement in cancer therapy [33]. We found that VD treatment inhibited apoptosis in *C. jejuni*-infected HT-29/B6 cells. This anti-apoptotic effect has also been reported in cholecalciferol-treated *C. jejuni*-infected IL-10^−/−^ mice [14], resulting in a decrease in antigen influx via the paracellular route.

*C. jejuni* first adhere to intestinal epithelial cells, then invade the epithelial layer and transmigrate via paracellular and transcellular pathways [34]. *C. jejuni* secrets a virulence factor—serine protease high-temperature requirement A (HtrA)—which has a critical role in transmigration, in that it cleaves epithelial junction proteins such as occludin and E-cadherin in Caco-2 cells, leading to enhanced bacterial invasion and paracellular transmigration [35,36,37]. We found that VD treatment decreased bacterial transmigration across *C. jejuni*-infected cell monolayers, which suggests that VD had an effect on the regulation of host cell signaling in addition to the VD-dependent cellular anti-bacterial mediators. The ability of VD to decrease *C. jejuni* transmigration has important functional implications, as it will tend to minimize both immune activation and the secondary deleterious effect of pro-inflammatory cytokines on barrier function (sustaining the ‘leaky gut’). In practical terms, this should result in a milder disease outcome.

### 3.2. C. jejuni Interference with the Vitamin D Pathway

Based on bioinformatic predictions from IPA of colon mucosae from *C. jejuni*-infected patients, the VD pathway showed overall inhibition of its activation state. This led us to hypothesize that *C. jejuni* infection might interfere with the VD pathway, and that VD supplementation might ameliorate *C. jejuni*-induced epithelial barrier defects. Evidence that VD has a protective effect on the intestinal epithelium in campylobacteriosis came from our preclinical intervention study in IL-10^−/−^ mice, which demonstrated that peroral administration of cholecalciferol reduced the severity of inflammation during acute *C. jejuni* infection [14]. Furthermore, *C. jejuni* infection inhibited the immune function mediators IL-1β, sCD14, β-defensin, and cathelicidin, which suggests a wider effect on the regulation of intestinal immunity. As defensins and cathelicidin are important components of the innate defense mechanisms at the surface of host gastrointestinal epithelia, we predicted from our data that *C. jejuni* would suppress innate immune responses for bacterial survival within the mucosa. However, contrary to our prediction, increased defensin and cathelicidin was reported in *C. jejuni*-infected cultured monolayers of Caco-2 or HT-29 cells [38]. In addition, a study with *Mycobacterium tuberculosis* demonstrated that treatment with VD increased the level of mRNA encoding protective cathelicidin [39], and the ability of VD to enhance the expression of antimicrobial cathelicidin has been reviewed in detail elsewhere [40].

CYP27B1 (25-hydroxyvitamin D-1-α-hydroxylase) is an important regulator of VD mechanism, which not only converts cholecalciferol to biologically active vitamin D3 (calcitriol) but also indirectly regulates antimicrobial responses in the gastrointestinal tract by inducing the expression of, for example, cathelicidin [41,42]. In our study, CYP27B1 protein expression tended to increase in *C. jejuni*-infected cells compared to uninfected control cells. Data generated in cytokine-stimulated PBMCs support this finding, with enhanced CYP27B1 expression in response to stimulation by IFN-γ and LPS [43] and in human monocytes when exposed to pathogens [44]. 

Immunostaining of VDR within its complex with RXR indicated that *C. jejuni* interfered with the VDR pathway. After *C. jejuni* infection, VDR signals appeared no longer restricted to the nuclei and were present increasingly in the cytosol. A change in VDR localization in colonic epithelial cells has also been reported during *Salmonella* infection [19]. As *C. jejuni* lacks enterotoxins, its intracellular appearance in the intestinal epithelial cells might directly influence the VDR/RXR complex, supposable via cytoskeleton interactions or via pro-inflammatory pathways such as NF-κB, as shown for *Salmonella* [13,19]. We found that VD treatment reduced the level of heterodimers in the cytoplasm, as described in previous studies [20]. In addition, the VDR/RXR complex accumulates in the cytosol after *C. jejuni* infection, probably as a result of the effect of RXR on the distribution of VDR [20], which supports the view that downstream gene expression is negatively affected. Thus, beneficial antimicrobial and immune strengthening downstream target genes, and therefore genes involved in the protective functionality of the VD pathway are inhibited during *Campylobacter* infection, but can be rescued by therapeutic doses of VD.

In conclusion, *C. jejuni* infection suppresses VD signaling pathways, which may be part of the explanation for the rogue host response seen in campylobacteriosis. Nevertheless, administration of the pre-hormone VD protects against *C. jejuni*-induced epithelial barrier dysfunction in vitro and in vivo. VD should therefore be considered as a potentially useful therapeutic supplement, when combined with other barrier-protective, immunomodulatory, and anti-inflammatory compounds, such as curcumin or resveratrol [3,45,46], for prevention and treatment of acute and chronic gastrointestinal disorders.

## 4. Materials and Methods

### 4.1. Cell Culture and Treatment of the Cells

The human colon epithelial cell line HT-29/B6-GR/MR [47] was cultured as described previously [3]. Briefly, cells were grown in RPMI 1640 medium (Sigma Aldrich, St. Louis, MO, USA) supplemented with 10% fetal calf serum (FCS; Gibco, Carlsbad, CA, USA), 1% penicillin/streptomycin (Corning, Wiesbaden, Germany), G418 (300 µg/mL; Invitrogen, Carlsbad, CA, USA), and hygromycin B (200 µg/mL; Biochrom GmbH, Berlin, Germany) at 37 °C in a humidified 5% CO_2_ atmosphere. Cells were passaged once a week and seeded on Millicell PCF filter membranes with a pore size of 3 µm and an effective growth area of 0.6 cm^2^ (Merck Millipore, Billerica, MA, USA). Seven days after seeding, confluent cell monolayers were ready for use (TER 600–900 Ω·cm^2^). One day before infection, cells were washed twice with antibiotic-free cell culture medium supplemented with heat-inactivated FCS. Cells were pre-treated with 200 nM of the biological active vitamin D calcitriol (VD; Cayman Chemicals, Ann Arbor, MA, USA) for 2 h from the apical and basal sides.

### 4.2. C. jejuni Cultivation and Experimental Infection In Vitro

*Campylobacter jejuni* wildtype (wt) 81–176 reference strain was cultured for two days on blood agar plates (Oxoid, Thermo Scientific, Waltham, MA, USA) under microaerobic conditions (5–10% O_2_, 10% CO_2_, 85% N_2_) generated with CampyGen gaspacks (Oxoid, Thermo Scientific, Waltham, MA, USA) in plastic jars at 37 °C. Bacteria were then cultured microaerobically in Mueller-–Hinton broth (200 rpm) for 2 h at 37 °C. Bacteria were centrifuged (2 min, 5000× *g*, 10 °C) and the optical density at OD_600_ adjusted to OD 1 with RPMI 1640 cell culture medium. Cultured cells were infected from the apical side with a multiplicity of infection (MOI) of 100. After infection, cells were incubated for 48 h at 37 °C under microaerobic conditions to encourage bacterial growth. Polarized MKN-28 cells were infected with *C. jejuni* wt strain NCTC11168 for up to 24 h with (50 nM to 200 nM) or without the addition of VD.

### 4.3. Generation of Secondary Abiotic IL-10^−/−^ Mice, Treatment and Experimental Infection In Vivo

IL-10^−/−^ mice with a C57BL/6j background were bred and housed under specific pathogen-free conditions in the animal facility of the Forschungseinrichtung für Experimentelle Medizin of Charité—Universitätsmedizin Berlin. Animal experiments were performed in the animal facility of Charité—Universitätsmedizin Berlin according to the European animal welfare guidelines (2010/63/EU), the ARRIVE guidelines and the German animal protection law (ethic committee: LAGeSo Berlin with the approval number G0172/16, 13 October 2016). The standard housing conditions, generation of secondary abiotic mice, treatment, and the infection of mice has been described previously [14]. To remove commensal gut microbiota, mice were transferred to sterile cages and treated for 8 weeks with an antibiotic cocktail containing ampicillin/sulbactam (1 g/L; Ratiopharm GmbH, Ulm, Germany), ciprofloxacin (200 mg/L; Bayer Vital, Leverkusen, Germany), imipenem (250 mg/L; MSD, Kenilworth, NJ, USA), metronidazole (1 g/L; Fresenius, Bad Homburg, Germany), and vancomycin (500 mg/L; Cell Pharm, Bad Vilbel, Germany) added to drinking water provided *ad libitum*. The antibiotic cocktail was replaced by cholecalciferol (25-OH-cholecalciferol; Sigma Aldrich, St. Louis, MO, USA) 4 days before oral infection. Cholecalciferol was dissolved in Tween 80 (0.2% *v*/*v*) and administered to mice via the drinking water. Mice received a daily cholecalciferol treatment dose of 500 µg per kg body weight (equivalent to 20,000 IU per kg) [14]. At days 0 and 1, mice were then perorally infected with 10^8^ colony forming units (CFU) of *C. jejuni* 81–176 in a volume of 0.3 mL PBS by gavage. Six days after infection, mice were sacrificed by isoflurane inhalation. Colon samples were removed and mounted in Ussing chambers in order to measure fluorescein fluxes (332 Da; Sigma Aldrich, St. Louis, MO, USA), as described previously [3].

### 4.4. Electrophysiological Methods

Transepithelial electrical resistance (TER) of cell monolayers was measured in vitro using chopstick electrodes under sterile conditions. TER was corrected for the resistance of an empty filter and calculated assuming a growth area of 0.6 cm^2^.

### 4.5. Epithelial Permeability Measurements

Measurements of fluorescein (332 Da) fluxes from apical to basolateral compartment were performed either in Ussing chambers for ex vivo samples or in 12-well plates for in vitro samples. Samples were taken every 15 min for one hour from the basolateral side, fluorescence measured spectrophotometrically (Tecan GmbH, Maennendorf, Switzerland), and permeability was calculated as flux/concentration differences.

### 4.6. Transmigration Assay

Polarized epithelial cell monolayers grown on 3 µm filter inserts were placed in 12-well plates and infected from the apical side with *C. jejuni* 81-176 or NCTC11168. A total of 25 µL of basolateral supernatant was removed in a time course after 2, 4, 8, and 24 h. The supernatant was diluted in cell culture medium and plated on to blood agar plates, which were incubated for 48 h under microaerobic conditions. The number of CFU was counted and multiplied with the factor of dilution to determine the number of transmigrated *C. jejuni.*

### 4.7. Western Blot

Changes in protein expression were quantified by Western blot analysis. Cells were washed twice with ice-cold PBS and lysed with whole cell lysis buffer containing 150 nM NaCl, 10 mM Tris buffer (pH 7.5), 0.5% Triton X-100, 1% SDS, and one tablet of Complete Protease Inhibitor (Roche AG, Mannheim, Germany) in a total volume of 10 mL. Cell lysis, protein quantification, and Western blot procedure were as described previously [3]. In brief, after cell lysis, cells were scraped carefully from the filters, transferred to reaction tubes, incubated 30–60 min on ice with regular vortexing, and centrifuged for 30 min (15,000*× g* at 4 °C). Proteins were detected on 12.5% polyacrylamide gels. Primary antibodies occludin (1:1000; Sigma Aldrich, St. Louis, MO, USA), claudin-1, -2, -4, -5, -7, -8 (1:1000; Invitrogen, Carlsbad, CA, USA), CYP27B1 (1:1000; Merck, Darmstadt, Germany), and β-actin (1:10,000; Sigma Aldrich, St. Louis, MO, USA) as loading control, were incubated slewing over night at 4 °C. Peroxidase-conjugated secondary antibodies to goat anti-rabbit IgG or goat anti-mouse IgG (Jackson ImmunoResearch, Ely, UK) were incubated slewing at room temperature for 2 h. Proteins were detected by incubating membranes in SuperSignal West Pico PLUS Stable Peroxide Solution (Thermo Scientific, Waltham, MA, USA), which were then visualized using a Fusion FX7 imaging system (Vilber Lourmat Deutschland GmbH, Eberhardzell, Germany), and analyzed by densitometry using Image Studio Lite version 5.2.

### 4.8. Immunostaining, Apoptosis Assay, and Confocal Microscopy

Epithelial cell monolayers were washed twice with PBS and fixed with 2% paraformaldehyde (PFA; Electron Microscopy Science, Hatfield, PA, USA) before staining to detect apoptosis, TJ proteins, nuclear factors, and effector proteins of the VD pathway as described previously [3]. The antibodies used were primary antibodies to occludin (1:100; Sigma Aldrich, St. Louis, MO, USA), claudin-4 (1:100; Invitrogen, Carlsbad, CA, USA), ZO-1 (1:100; BD Bioscience, Franklin Lakes, NJ, USA), VDR (1:100; Abcam, Cambridge, UK), RXR (1:100; Novus Biologicals, Littleton, CO, USA) as well as secondary antibodies conjugated to Alexa-Fluor 488 or 594 (1:500; Invitrogen, Carlsbad, CA, USA) with incubation for 1 h at 37 °C. Apoptotic events were detected using the TUNEL assay (In situ Cell Death Detection Kit, Roche, Mannheim, Germany) according to the manufacturer’s instructions. Nuclei were stained with 4′,6-diamidino-2-phenylindole (DAPI; 1:1000; Roche, Basel, Switzerland). Tight junction structure, subcellular protein distribution, and apoptotic cells were visualized by confocal laser-scanning microscopy (CLSM; Zeiss LSM780, Jena, Germany).

### 4.9. RNA Expression Analysis

RNA isolation from colonic samples from six control patients and four *C. jejuni*-infected patients, next-generation sequencing, and RNA-Seq expression analysis was performed and described previously [2]. Data were deposited in NCBI’s Gene Expression Omnibus (GEO ID GSE88710). Ingenuity Pathway Analysis software (IPA; Qiagen, Redwood, CA, USA) was used to analyze *C. jejuni*-dependent pathway activation.

### 4.10. Statistical Analysis

All data are presented as mean values ± standard error of the mean (SEM). Statistical analyses were performed with GraphPad Prism version 8.0 using Student´s *t*-test for comparison of two or one-way ANOVA comparing more than two groups with Bonferroni adjustment for multiple comparison. Data which were not normally distributed were analyzed using the nonparametric Mann–Whitney U-test. *p* < 0.05 was considered to be statistically significant.

## Figures and Tables

**Table 1:** Tight junction (TJ) protein expression change from control level (100%) in HT-29/B6-GR/MR cells after *C. jejuni* infection with or without addition of 200 nM vitamin D.

TJ Protein	*C. jejuni*	Vitamin D	Vitamin D *+ C. jejuni*
claudin-4	98 ± 9%	95 ± 12%	90 ± 10%
claudin-5	85 ± 13%	93 ± 27%	86 ± 12%
claudin-7	89 ± 15%	101 ± 11%	100 ± 14%
claudin-8	92 ± 15%	93 ± 17%	110 ± 17%
occludin	103 ± 18%	109 ± 16%	99 ± 16%

TJ: tight junction; one-way ANOVA with Bonferroni correction, all comparisons not significant.

## Data Availability

All generated data supporting the key findings are available in the manuscript. Raw data are available upon request. RNA-seq raw data are deposited at NCBI’s Gene Expression Omnibus under the GEO accession ID GSE88710.

## Abbreviations

CFU	colony forming unit
CLSM	confocal laser-scanning microscopy
DAPI	4′,6-diamidino-2-phenylindole
DSS	dextran sulfate sodium
FCS	fetal calf serum
HtrA	high-temperature requirement A
IBD	inflammatory bowel disease
IBS	irritable bowel syndrome
IFN	interferon
IPA	Ingenuity Pathway Analysis
LPS	lipopolysaccharide
MOI	multiplicity of infection
OD	optical density
RXR	retinoid X receptor
TER	transepithelial electrical resistance
TJ	tight junction
TNBS	2,4,6-trinitrobenzenesulfonic acid
TNF	tumor necrosis factor
TUNEL	TdT-mediated dUTP-biotin nick end labeling
VD	vitamin D
VDR	vitamin D receptor
ZO-1	zonula occludens-1
